# Protective effect of astaxanthin nanoemulsion on mammalian inner ear hair cells

**DOI:** 10.7717/peerj.15562

**Published:** 2023-09-08

**Authors:** Yuki Kobayashi, Kazuma Sugahara, Yosuke Takemoto, Junko Tsuda, Yoshinobu Hirose, Makoto Hashimoto, Hiroshi Yamashita

**Affiliations:** Department of Otolaryngology, Yamaguchi University Graduate School of Medicine, Ube, Yamaguchi, Japan

**Keywords:** Astaxanthin, Inner ear, Nanoformulation, Round window membrane, Hair cells

## Abstract

**Background:**

Aminoglycoside antibiotics are used for treating certain acute infections. However, these drugs cause ototoxicity by inducing inner ear hair cell death.

**Aims/Objectives:**

We investigated the protective effect of a nanoemulsion of the carotenoid astaxanthin on mammalian inner ear hair cells against neomycin-induced ototoxicity.

**Material and Methods:**

Dose-response relationship, quantification of hair cell loss, and reactive oxygen species production were assayed in response to neomycin with and without astaxanthin in cultured utricles of CBA/N mice. In addition, auditory brain response (ABR) and hair cell loss after exposure to the nanoformulation and loud noise were examined in vivo in guinea pigs.

**Results:**

Astaxanthin suppressed neomycin-induced reduction of hair cells by reducing the production of hydroxy radicals. Furthermore, hair cell loss in the second rotation of the cochlea was significantly lower in the astaxanthin group than in the noise-only group.

**Conclusions and Significance:**

The blood-labyrinth barrier limits the successful delivery of drugs for inner ear complications. However, in the nanoemulsion form, astaxanthin can penetrate the round window (fenestra ovale) membrane, enabling topical administration. Thus, astaxanthin nanoemulsion could be useful in treating ototoxicity in individuals with inner ear complications.

## Introduction

Inner ear hair cell death induced by aminoglycoside drugs is known to be related to the production of reactive oxygen species (ROS) and stress kinases (Priuska & Schacht, 1995). Drug supplements, vitamin E, CoQ10, and quercetin reportedly have protective effects on inner ear hair cells (Hirose et al., 2016; Sugahara et al., 2014). Carotenoids might have beneficial effects as well since they are known to reduce oxidative stress and cell death. Astaxanthin is a type of carotenoid, and dietary supplements and cosmetics containing astaxanthin are widely available. The uptake of astaxanthin by biological tissues is a concern because of its lipid-soluble nature. In 2007, Fujifilm Corporation (Japan) developed an astaxanthin nanoemulsion that can diffuse into water, be delivered to living tissues, and be transferred *via* blood (Harada et al., 2017).

Few studies have examined the protective effects of astaxanthin nanopreparations on the inner ear (Horiike, Shimogori & Yamashita, 2004; Takemoto et al., 2018). In the present study, we evaluated the protective effect of astaxanthin nanoemulsion on mammalian inner ear hair cells against neomycin-induced ototoxicity and acoustic damage *in vitro* and *in vivo*.

## Materials & Methods

### Preparation of astaxanthin nanoemulsion

Astaxanthin nanoemulsion (Fujifilm Company, Tokyo, Japan), dissolved in a vehicle and in an experimental medium before the initiation of cell culture, was used in this study.

Three kinds of astaxanthin concoctions, each at concentrations from 100 nM–1 mM, were prepared for absorbance measurement: astaxanthin nanoemulsion diluted with water, astaxanthin bulk powder (Sigma Chemical Co., St. Louis, MO, USA) dissolved in absolute ethanol, and astaxanthin dissolved in dimethyl sulfoxide (DMSO), and further dissolved in water. The absorbance at 480 nm of each of the five concentrations (diluted 10-fold from 10 mM to 1 nM) of the three astaxanthin preparations was measured. The astaxanthin concentration of each sample was calculated from the absorbance.

### *In vitro* experiments

Male CBA/N mice (4–6 weeks old) with normal Preyer’s reflexes were obtained from Kyushu Animal Company (Kumamoto, Japan). All animals were anesthetized with an overdose of pentobarbital, decapitated immediately, and the temporal bones were removed quickly. Vestibular organs were dissected in basal Eagle’s medium (Invitrogen, Carlsbad, CA, USA) supplemented with Earle’s balanced salt solution (Invitrogen) (2:1, v/v), and the utricles were isolated. They were placed in the culture medium comprising basal Eagle medium supplemented with Earle’s balanced salt solution (2:1, v/v) and 5% fetal bovine serum (Invitrogen).

### Dose–response relationship

Utricles were divided into five groups as follows: control, neomycin (2 mM, Sigma Chemical Co.), neomycin+astaxanthin (1 µM), neomycin+astaxanthin (10 µM), and neomycin+astaxanthin (1–100 µM). In the control group, only the vehicle was added to the medium. In the neomycin group, neomycin was added to the culture at a concentration of 2.0 mM. In the neomycin+astaxanthin groups, in addition to 2 mM neomycin, astaxanthin was added at 1 µM, 10 µM, and 100 µM, respectively. The utricles were incubated for 1 h before exposure to neomycin.

All free-floating utricles were incubated for 24 h at 37 °C in 24-well tissue culture plates in a 5% CO_2_ and 95% air environment. After all culture protocols, utricles were fixed with 4% paraformaldehyde (PFA) for 1 h at room temperature. Otoconia from the fixed utricles were gently removed using a stream of phosphate-buffered saline (PBS) applied through a syringe and a 28G needle. After the samples were rinsed with PBS, they were subsequently used in assays.

### Immunohistochemistry for hair cell labeling

Fixed utricles were incubated overnight at 4 °C in blocking solution (1% bovine serum albumin, 0.4% normal goat serum, 0.4% normal horse serum, and 0.4% Triton X-100 in PBS). A monoclonal antibody against calmodulin (Sigma) was used to label the hair cells. Samples were incubated in primary antibody solution (1:150 in blocking solution) overnight at 4 °C.

Specimens were washed using the blocking solution and then incubated with the following secondary antibody (Alexa 488-conjugated goat anti-mouse IgG; Molecular Probes, Eugene, OR, USA) diluted 1:500 in blocking solution. Utricles were rinsed using blocking solution, mounted in Vectashield^®^ (Vector Laboratories, Newark, CA, USA), and coverslipped.

### Quantification of residual sensory hair cells

Utricles were observed using a fluorescence microscope (XF-EHD2; Nikon, Tokyo, Japan) to evaluate the survival of hair cells. Calmodulin-positive cells were indicative of survival. In each utricle, labeled hair cells were counted within two randomly selected squares of 20 µm ×20 µm, and eight hair cell counts were averaged to calculate hair cell density. At least six utricles were examined in each condition. All data were expressed as mean ± standard error and analyzed using StatView version 5.0J for Macintosh (SAS Institute Inc., Cary, NC, USA). The Mann–Whitney U test was used to compare hair cell densities and significant values. A *P* value <0.05 was considered statistically significant.

### Immunohistochemistry for 4-hydroxy-2-nonenal production

Using a method described previously (Sugahara et al., 2014), 4-hydroxy-2-nonenal (4-HNE) production was investigated to evaluate the production of ROS. The samples were fixed using 4% PFA after dissection, and utricles were incubated in a 1:100 dilution of anti-4-HNE mouse monoclonal antibody (OXIS International, Inc., Portland, OR, USA) overnight in a refrigerator (4 °C). Samples were rinsed in blocking solution and incubated with Alexa 488-conjugated goat anti-mouse IgG and Texas red-conjugated phalloidin (1:100, Sigma) for 4 h at room temperature. The fluorescence intensity of the immunohistochemical staining was evaluated using the image analysis software ImageJ. Cultured utricles were divided into three groups: control (using normal medium), neomycin, and neomycin+astaxanthin. Six samples per group were used for the experiment. The average of the fluorescence intensities derived from the control group was defined as 20, and the intensities in the other groups were shown by the relative value.

### *In vivo* experiments

Male guinea pigs (4–24 weeks old) obtained from the Kyushu Animal Company were anesthetized by intraperitoneal administration of a mixture of medetomidine (0.3 mg/0.3 mL/kg), midazolam (4 mg/0.8 mL/kg), and butorphanol (5 mg/mL/kg) before the auditory brain stem response (ABR) examination or dissection.

The right ears of guinea pigs were defined as the noise-only group, while the left ears were the astaxanthin group (*n* = 7 each). The temporal bone was exposed *via* a postauricular incision. The mastoid bulla was opened using a 4-mm diamond burr to allow the visualization of the round window. Hyaluronic acid drops (1% at 10 mg/mL, Japan) were applied on the round window membrane (RWM) on both sides for 10 min before the administration of the drug. On the left side, an astaxanthin emulsion (100 µl nanoformulation with a particle size of 60 nm and astaxanthin content of 0.76 wt%) was applied on the RWM. Similarly, only the solvent (100 µl nanoformulation with a particle size of 55 nm) was applied to the right RWM. The mastoid bulla hole was closed with dental cement (GC Fuji, GC Co., Tokyo, Japan), and the skin incision was closed.

### Transfer of the drug into the cochlea

To confirm the transfer of the drug into the cochlea, perilymph was collected from the cochlea *via* the circular lamina on the day after the administration of the drug, and the spectra of the four samples were obtained using Nanodrop (Thermo Fisher Scientific K.K., Tokyo, Japan). The absorbance at 480 nm was also assessed.

### Sound exposure

Each guinea pig was anesthetized as described above, and a speaker was placed 10 cm above the center of the head in an anechoic room. The sound pressure was measured in flat mode using a sound-level meter (NA-60; Rion, Tokyo, Japan) at the height of both ear canals to produce a loud sound (130 dB SPL, octave band noise centered at 4 kHz).

The first auditory brainstem response (ABR) test confirmed that the animals used did not show an increase in threshold and that there was no difference between the left and right ears (Gourévitch & Edeline, 2011). The sound exposure was started 24 h after drug administration. Animals were exposed to loud sound (130 dB SPL, octave band noise centered at 4 kHz) for 3 h.

Before and 7 days after the acoustic exposure, ABR tests were performed to evaluate hearing ability. In the ABR test, a platinum needle electrode was inserted under the skin of the animal and the reaction was recorded using the test side as a cathode, the other side as an anode, and the trunk under the body as the ground electrode. The tube was connected to earphones, inserted into the ear canal, and exposed to a stimulus tone set at 4, 8, 16, and 32 kHz tone bursts for 0.8 s at 0.2-s intervals for a total of 500 stimuli. A signal processor (RZ6 Multi-I/O Processor; Tucker-Davis Technologies) was used as the ABR recording device. The threshold was the value that could be measured with the lowest stimulus having a waveform between 3 and 5 waves.

### Quantification of hair cell loss

After guinea pigs were anesthetized using an intraperitoneal injection of a mixture of medetomidine (0.3 mg/0.3 mL/kg), midazolam (4 mg/0.8 nL/kg), butorphanol (5 mg/mL/kg), and saline (2.9 mL/kg), the head was promptly decapitated and the temporal bone was removed. The cochlea was extracted from the temporal bone and fixed with 4% PFA for 24 h at room temperature. The organ of Corti was excised, permeated with 0.3% Triton X-100 for 10 min, and stained with fluorescein isothiocyanate-conjugated phalloidin (1 µg/mL PBS; Sigma) for 1 h. Samples were washed with PBS and observed using fluorescence microscopy. The outer hair cells between 45% and 70% of the distance from the apex to the basal rotation (second rotation of the cochlea) were observed. The reason for observing the outer hair cells in this region is that the outer cochlea forms the strongest obstacle to rotation because of the octave band noise centered at 4 kHz (Viberg & Canlon, 2004).

### Statistical analysis

Statistical analysis was performed using the Mann–Whitney U test in Excel Stat4 for Windows (Microsoft, Tokyo, Japan), and *P* < 0.05 was considered significant. All data were shown as mean ± standard error.

The experimental protocol was reviewed and approved by the Committee for Ethics on Animal Experiments at the Yamaguchi University School of Medicine (43002). This study was conducted in accordance with the guidelines of the Japanese Federal Law (No. 105) and Notification No. 6 of the Japanese Government.

## Results

### Absorbance measurement

**Figure 1:**
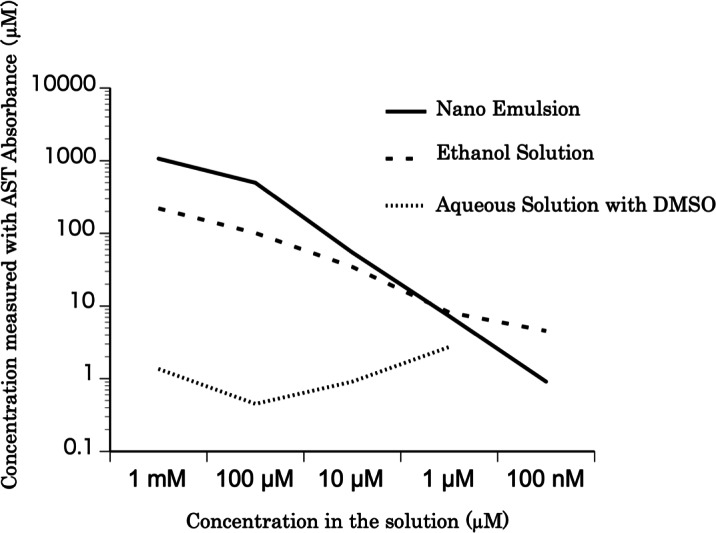
The concentration of the astaxanthin nanoemulsion measured from the absorbance. The concentration of the astaxanthin nanoemulsion measured from the absorbance was higher than that of the DMSO solution. The astaxanthin nanoemulsion was found to produce a high-concentration aqueous solution of astaxanthin.

The relationship between the astaxanthin concentration calculated based on the absorbance values and the astaxanthin concentrations in the solutions is shown in Fig. 1. The concentration of the nanoemulsion was similar to that of the ethanol solution. However, the concentration of astaxanthin in the DMSO solution was lower than that of astaxanthin in the ethanol solution. Thus, the astaxanthin nanoemulsion can produce a high-concentration aqueous solution of astaxanthin than astaxanthin bulk powder.

### *In vitro* experiments

#### Dose-relationship

**Figure 2:**
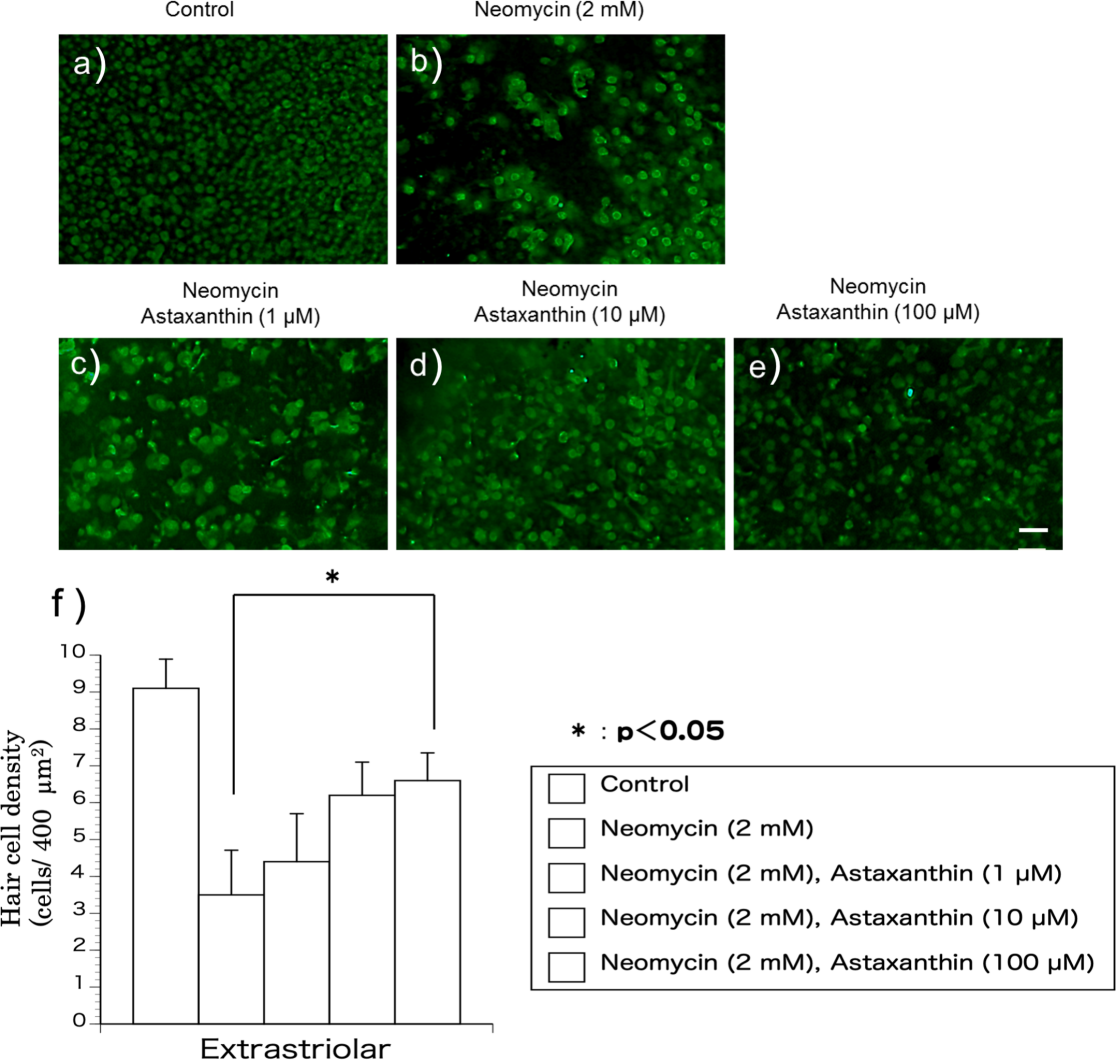
(A–F) The protective effect of astaxanthin against hair cell loss caused by aminoglycoside. Neomycin caused hair cell damage compared with the control. Astaxanthin (100 µM) protected hair cells against aminoglycoside ototoxicity (^∗^*p* < 0.05). (Scale bar in *e* = 10 µm).

The utricles were cultured with neomycin (2 mM) and astaxanthin (1–100 µM) for 24 h to evaluate the effect of astaxanthin on the survival rate of hair cells in the presence of neomycin. After exposure to the reagents, the utricles were fixed for 24 h. To detect residual hair cells, calmodulin was immunolabelled (Figs. 2A–2E). In the medium containing neomycin, the hair cell densities were reduced after 24 h. In contrast, more hair cells survived in the medium containing both neomycin and astaxanthin. The densities of cultured hair cells are shown in Fig. 2F. The reduction in hair cells induced by neomycin was suppressed by astaxanthin. The effect of astaxanthin showed a dose–response relationship. Therefore, astaxanthin at a concentration of 100 µM could significantly protect hair cells against the cytotoxicity of neomycin.

#### Immunohistochemistry for 4-HNE production

**Figure 3:**
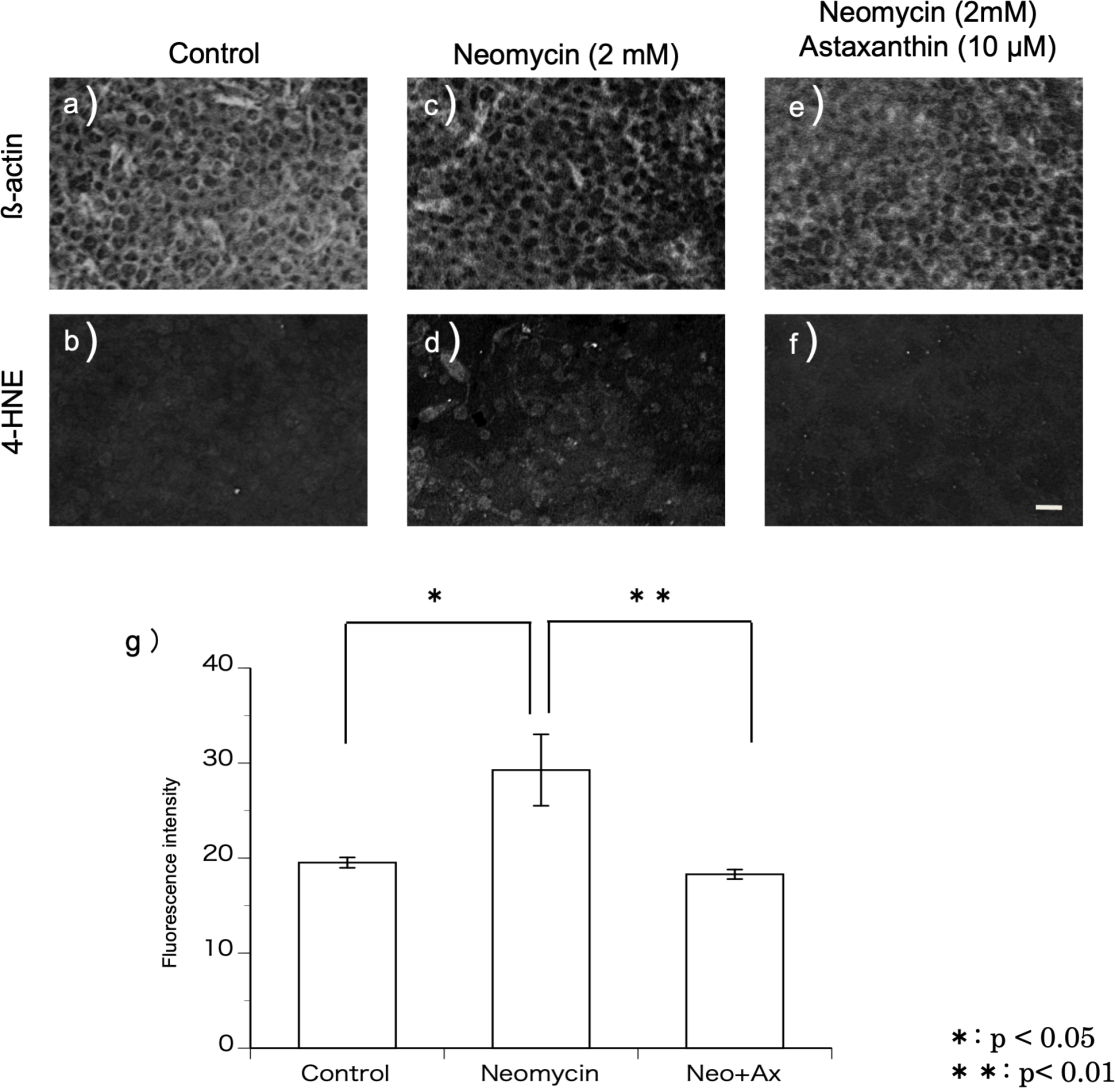
(A–G) Fluorescence intensity of 4-HNE. The fluorescence intensity of 4-HNE was significantly stronger in the utricles cultured with neomycin than in those cultured without neomycin. The presence of astaxanthin (10 µM) protected against oxidative stress (^∗∗^*p* < 0.01). (Scale bar in *f* = 10 µm).

4-HNE is a metabolic product of the hydroxy radical. Immunohistochemistry was performed using the antibody against 4-HNE to detect the production of hydroxy radicals (Figs. 3A–3F). In this study, utricles cultured for 12 h were used because 2 mM neomycin reduced the hair cells 24 h after exposure, as shown above. To highlight the hair cell layer, *β*-actin was labeled with phalloidin conjugated with Texas red. A fluorescence microscope was used to focus on the hair cell layer. Hair cells with 4-HNE were not observed in the utricles cultured for 12 h without neomycin. In the utricles cultured with 2 mM neomycin, numerous hair cells with 4-HNE were observed. In the utricles cultured with both neomycin and astaxanthin for 12 h, 4-HNE signals were not observed. These results indicated that astaxanthin suppressed the production of hydroxy radicals induced by neomycin.

The fluorescence intensity of the immunohistochemical staining was evaluated (Fig. 3G). The fluorescence intensity derived from 4-HNE was significantly stronger in the utricles cultured with neomycin than in those without neomycin. Therefore, the presence of astaxanthin significantly inhibited fluorescence intensity.

### *In vivo* experiments

#### Transfer of the drug into the cochlea

**Figure 4:**
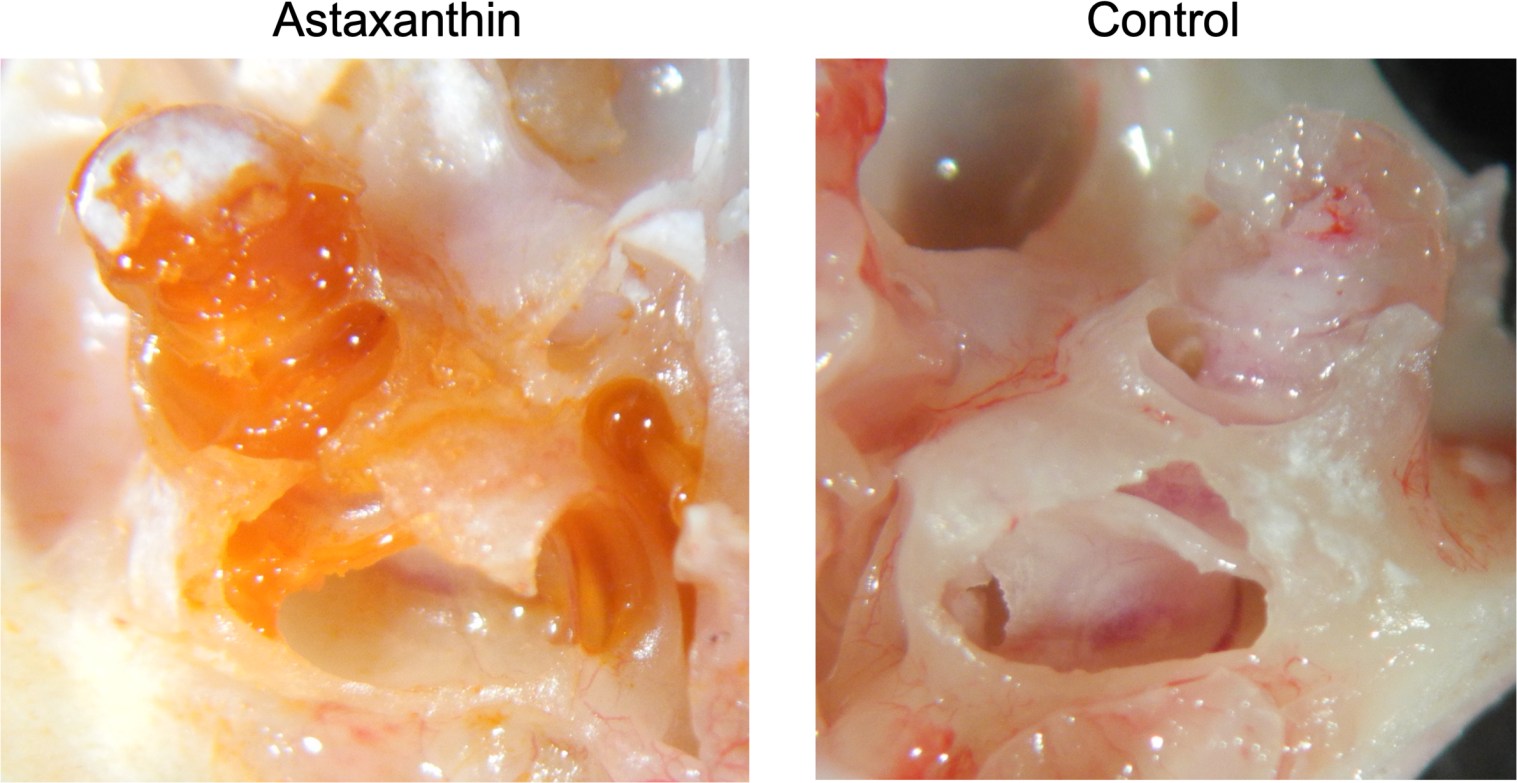
Presence of the drug in the cochlea. In the astaxanthin group, a change in red color was observed in the cochlea, especially the basal layer. No color change was observed in the control group, to which only the solvent was administered.

Twenty-four hours after the administration of astaxanthin, guinea pig cochleae were excised, and the color of the internal lymph was observed. In the astaxanthin group, red color similar to astaxanthin was observed particularly in the basal turn layer of the cochlea. No color change was observed in the control group, which received only the solvent (Fig. 4). The average spectrum of samples collected from the internal lymph at 480 nm was 0.6425, which corresponded to an astaxanthin concentration of 292.05 µM. The difference of average spectrum between astaxanthin group and the control group was 0.233, which corresponded to an astaxanthin concentration of 106.045 µM.

#### ABR threshold before and after sound exposure

**Figure 5:**
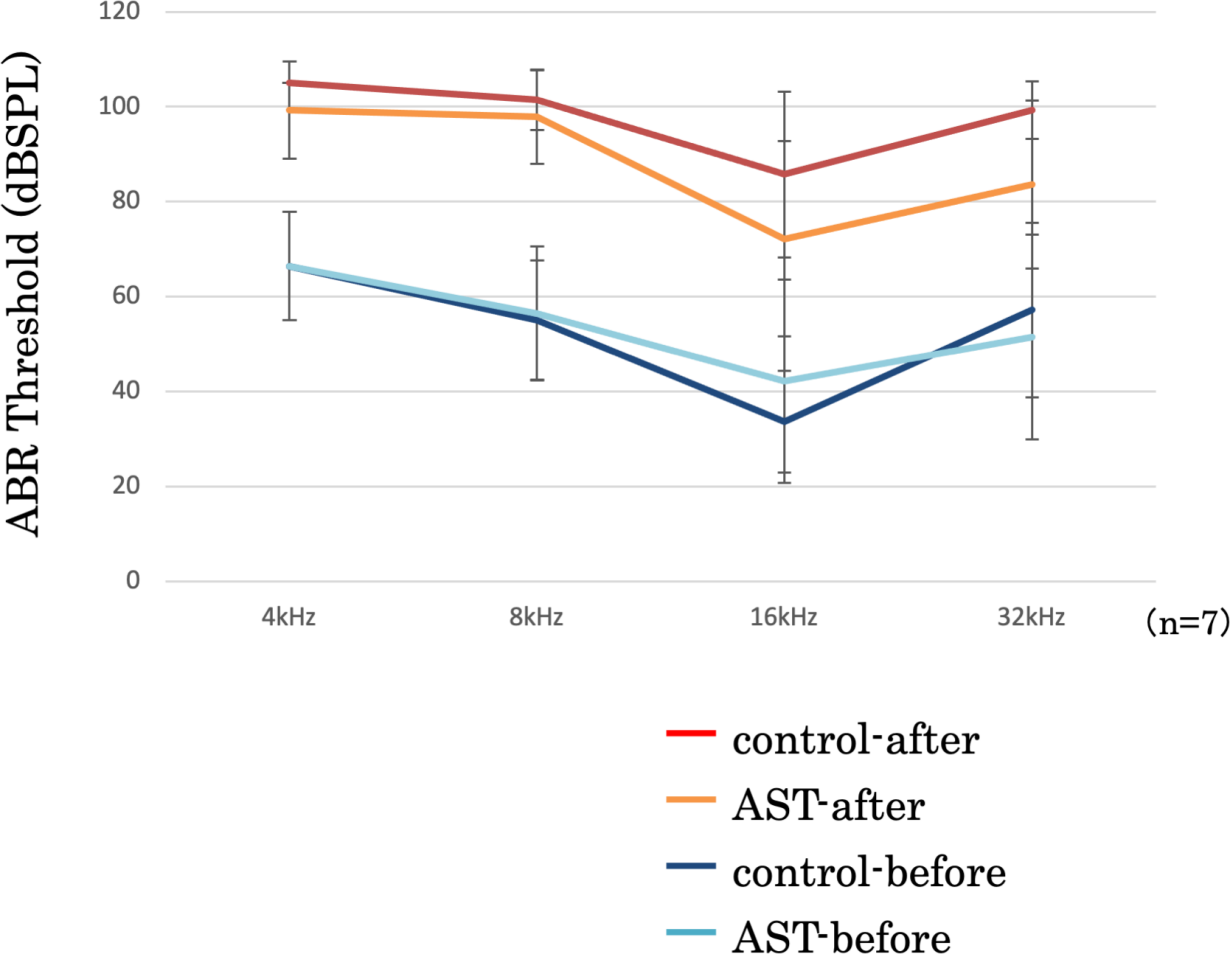
Suppression of the ABR threshold shift. ABR thresholds of all frequencies increased after sound exposure. However, the threshold shift was smaller in the astaxanthin group than in the control group at 16 kHz. ^∗^*p* < 0.05.

The threshold shifts were measured at 4-, 8-, 16-, and 32-kHz stimuli (Fig. 5). In this figure, the vertical axis represents the thresholds, measured in decibels (dB) of sound pressure level (dBSPL) before and after exposure to sound. The error bars indicate ±1 S.E.M. The threshold shifts before and after sound exposure were compared between the astaxanthin and control groups. There was no significant difference at 4, 8, and 32 kHz. Though the ABR threshold shift at 16 kHz appeared to be suppressed in the astaxanthin group, there was no significant difference between the two groups seven days after exposure to sound.

#### Quantification of hair cell loss

**Figure 6:**
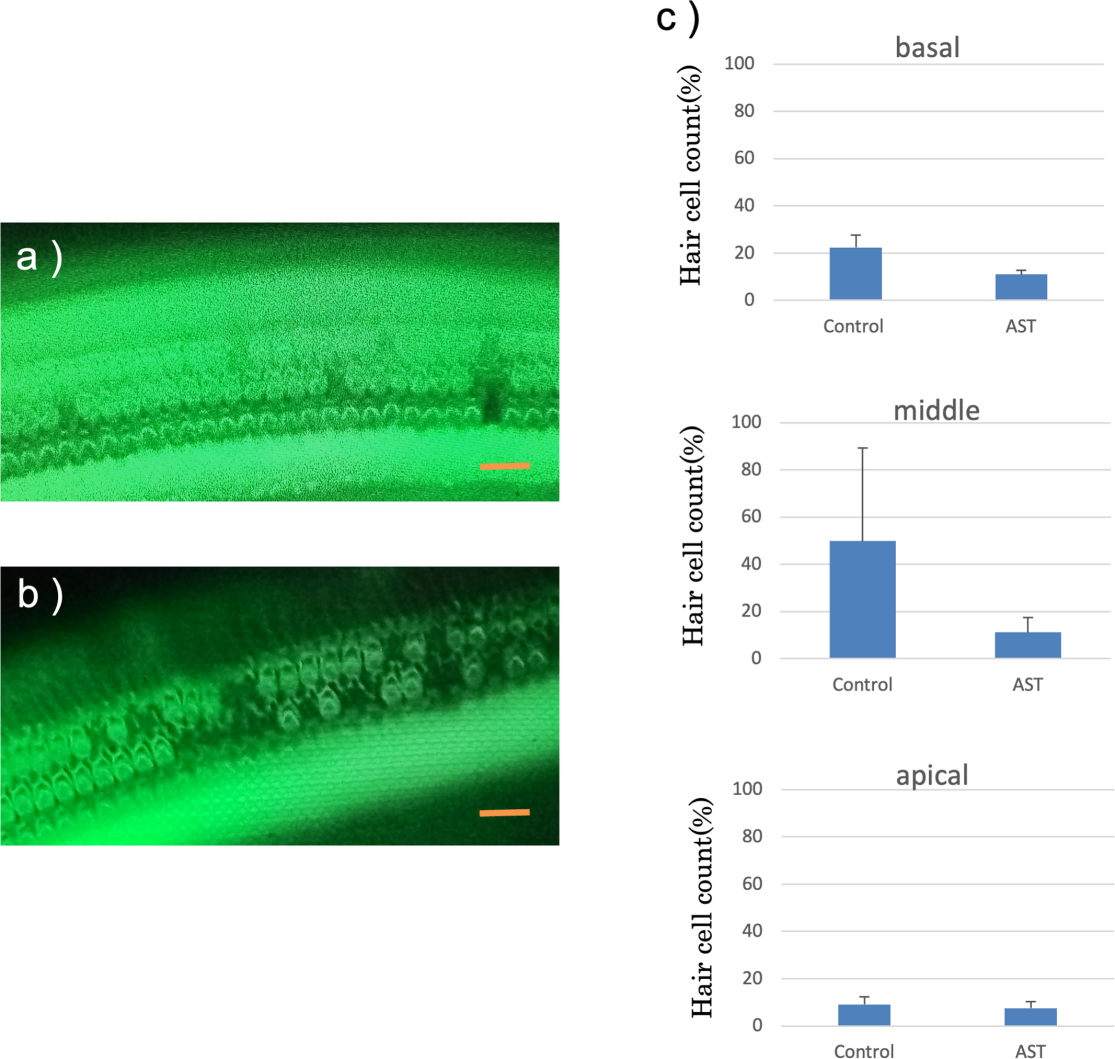
Surface structure of the organ of Corti. (A) AST-treated ear, (B) solvent-treated ear. Fluorescence microscopy revealed that the outer and inner hair cells in the astaxanthin-treated side looked almost normal 7 days after sound exposure. (Scale bars = 20 µm). (C) The protective effect of astaxanthin against sound exposure-induced outer hair cell loss. There was less hair cell loss in the astaxanthin group than in the vehicle control group in the middle layer of the cochlea.There was no significant difference in the inner hair cell loss rates.

The features of the inner hair cells and three rows of outer hair cells seven days after exposure to sound are shown in Figs. 6A and 6B. The rates of hair cell survive in the basal, middle and apical rotation of the cochlea are shown in Fig. 6C. More hair cells survived in the astaxanthin group than in the control group. The rates of hair cell loss were lower in the astaxanthin group than in the noise-only group in the middle of the cochlea (Fig. 6C). In contrast, inner hair cells were barely reduced in both the groups, and there was no significant difference in the rates of inner hair cell loss.

## Discussion

There is strong evidence that the production of ROS is closely associated with inner ear disorders (Priuska & Schacht, 1995). The aminoglycoside–iron complex-induced mechanism of ROS formation is well studied (Thomas et al., 2009). This complex enhances iron-catalyzed oxidation and promotes the formation of ROS. While aminoglycosides (such as neomycin) are believed to activate ROS as well as stress kinases and contribute to hair cell damage, the presence of free radical scavengers has been reported to reduce such damage. When aminoglycosides combine with iron salts, astaxanthin as an antioxidant agent suppresses the oxidation reaction of singlet oxygen. Singlet oxygen is particularly toxic among the ROS because of its role in generating lipid peroxide. Astaxanthin as an antioxidant agent suppresses the oxidation reaction of singlet oxygen, reduces oxidative stress and cell death (Goto et al., 2001; Lakey-Beitia et al., 2019).

Many carotenoids have been reported to cross the blood–brain barrier (Lakey-Beitia et al., 2019; Taksima et al., 2019). Astaxanthin has also been reported to cross the blood–brain and blood–retinal barriers and penetrate the skin (Uragami et al., 2012). The beneficial effects of astaxanthin include inhibition of diabetic complications (Zhou et al., 2015), suppression of eye diseases (Dong et al., 2013), cancer prevention (Cicero et al., 2019), and anti-fatigue action (Imai et al., 2018). In an earlier study, we reported that oral administration of astaxanthin nanoformulations resulted in a higher percentage of hair cells remaining in the lateral organs of zebrafish treated with neomycin than that in the group without astaxanthin treatment (Takemoto et al., 2018). Similarly, in the present study, the presence of astaxanthin nanoemulsion significantly cut down the rate of hair cell reduction in cultured mouse utricles. This implies that astaxanthin reduced neomycin-induced sensory hair cell death in the mammalian inner ear epithelium. Thus, astaxanthin nanoemulsion can be used as a protective agent for the inner ear.

Drug permeability in the inner ear is hindered by a blood-labyrinth barrier and low blood circulation, making drug access to this part of the ear difficult. To overcome these limitations, intratympanic injection is generally used for local administration of a drug to the inner ear. In a previous study, we reported that topical administration of edaravone suppressed streptomycin-induced vestibulotoxicity much better than systemic administration of the same (Horiike, Shimogori & Yamashita, 2004).

Nanoparticles such as polylactic-*co*-glycolic acid have been shown to cross the RWM (Zhang et al., 2018). Therefore, nanopharmaceutical formulations are believed to penetrate the RWM easily, making them suitable for topical administration (Inagaki et al., 2016). In this study, a nanoformulation was used topically. A color change was observed in the perilymph after the administration of the drug, and astaxanthin was identified based on its absorbance. The relevance of this approach in clinical applications looks promising. Furthermore, retention in the tympanic chamber and a sustained release effect are required for a drug to be efficacious (Horiike, Shimogori & Yamashita, 2004). Therefore, the route of administration needs to be investigated further in future studies.

We have used two strains of animals in this study. Mice are suitable for experiments with cultured utricles, and guinea pigs are suitable for experiments exposing round windows. Results could be obtained using animals suitable for the particular types of experiments.

The ABR threshold should ideally have been measured between the treatment and acoustic exposure. We have not measured the thresholds in this way because guinea pigs are stress-sensitive. Therefore, the effect of treatment on the ABR threshold could not be ruled out. To further confirm this effect, we performed surgery and administered the solvent in the control right ear. In addition, we have confirmed that the nanoemulsion of astaxanthin did not induce ototoxicity in the *in vitro* experiment in this study.

It is desirable to use the same model of inner ear damage caused by aminoglycosides in *in vivo* experiments as *in vitro*. However, it is difficult to administer aminoglycosides in large enough doses to cause inner ear damage because the survival rate of experimental animals is reduced. Therefore, an acoustic injury model was used as an *in vivo* model that can stably produce inner ear damage. The following factors are intricately involved in acoustic damage: ROS generation related to impaired blood flow (Yamashita et al., 2004), loss of many afferent synapses (Kujawa & Liberman, 2009) and synaptic damage due to excessive glutamate release (Pujol et al., 1990), tissue damage due to inflammatory cytokines (Fujioka et al., 2006), and multiple other factors. A limitation for this experiment is that we did not have the same experimental conditions among *in vitro* and *in vivo* experiments.

## Conclusions

We found that the astaxanthin nanoformulation suppressed damage to inner ear hair cells. In further studies, effective approaches for administering the drug will be explored for its stable transfer to the inner ear. This approach has considerable potential for devising clinical applications to prevent inner ear damage.

##  Supplemental Information

10.7717/peerj.15562/supp-1 Supplemental Information 1 Data for Fig. 1

10.7717/peerj.15562/supp-2 Supplemental Information 2 Data for Fig. 2

10.7717/peerj.15562/supp-3 Supplemental Information 3 Data for Fig. 3

10.7717/peerj.15562/supp-4 Supplemental Information 4 Data for Fig. 5

10.7717/peerj.15562/supp-5 Supplemental Information 5 Original photo of Fig. 4, control

10.7717/peerj.15562/supp-6 Supplemental Information 6 Original photo of Fig. 4, astaxanthin

10.7717/peerj.15562/supp-7 Supplemental Information 7 Original photo of Fig. 6, AST-treated ear

10.7717/peerj.15562/supp-8 Supplemental Information 8 Original photo of Fig. 6, solvent-treated ear

10.7717/peerj.15562/supp-9 Supplemental Information 9 Outer hair cell loss after intense noise exposure Raw data of the rate of outer hair cell loss in the middle layer of the cochlea was shown.

10.7717/peerj.15562/supp-10 Supplemental Information 10 Hair cell loss after intense noise exposure Raw data of the rate of inner and outer hair cell loss in all layers of the cochlea was shown.

## Additional Information and Declarations

### Data Availability

The following information was supplied regarding data availability: The raw measurements are available in the Supplementary Files.
