# Supplementary figures and images for: Protective effect of astaxanthin nanoemulsion on mammalian inner ear hair cells
Source: PeerJ. 2023 Sep 8;11:e15562. doi: 10.7717/peerj.15562 (PMC10494832; doi:10.7717/peerj.15562)

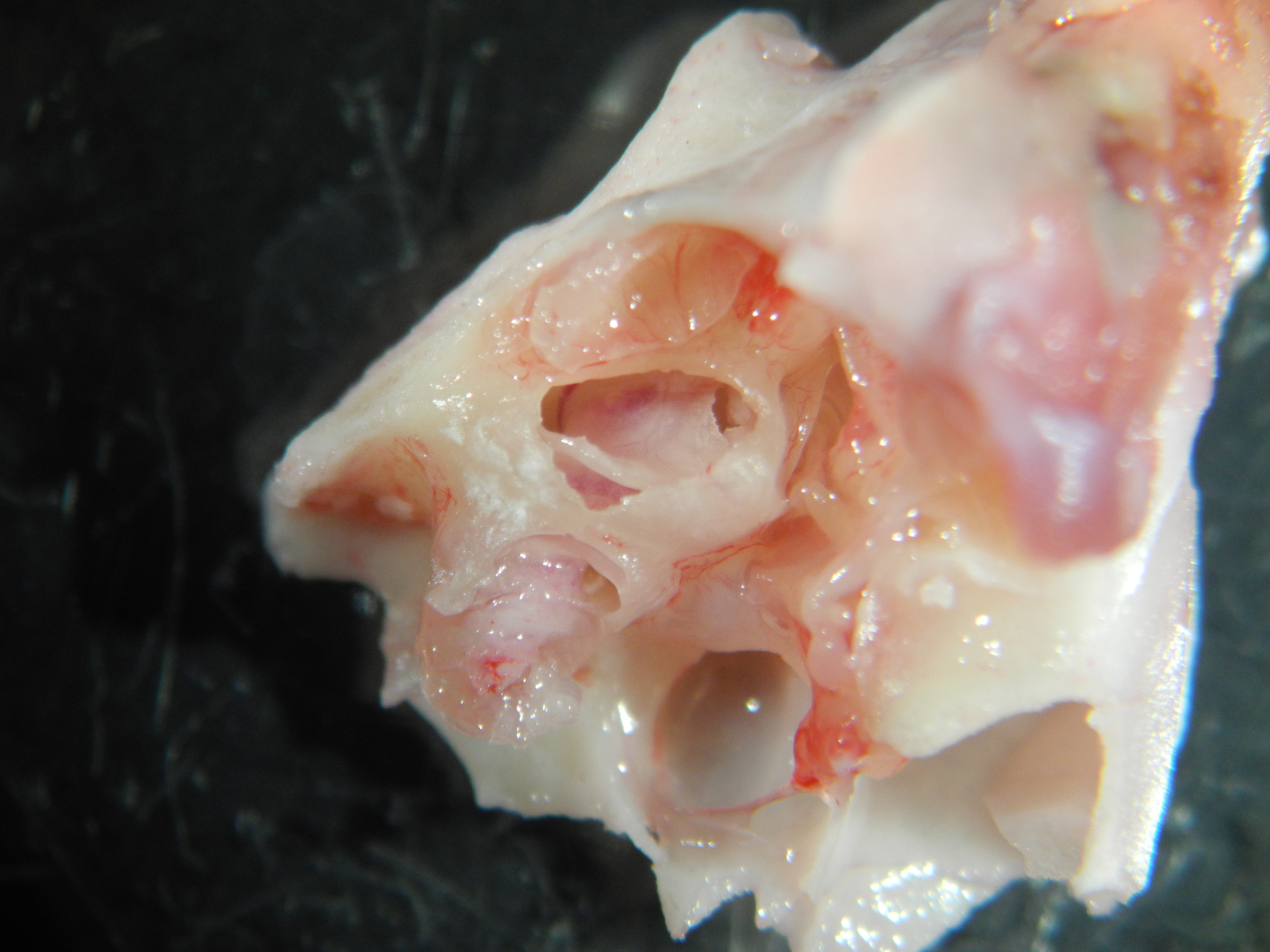

Supplement: Supplemental Information 5 [file peerj-11-15562-s005.jpg]

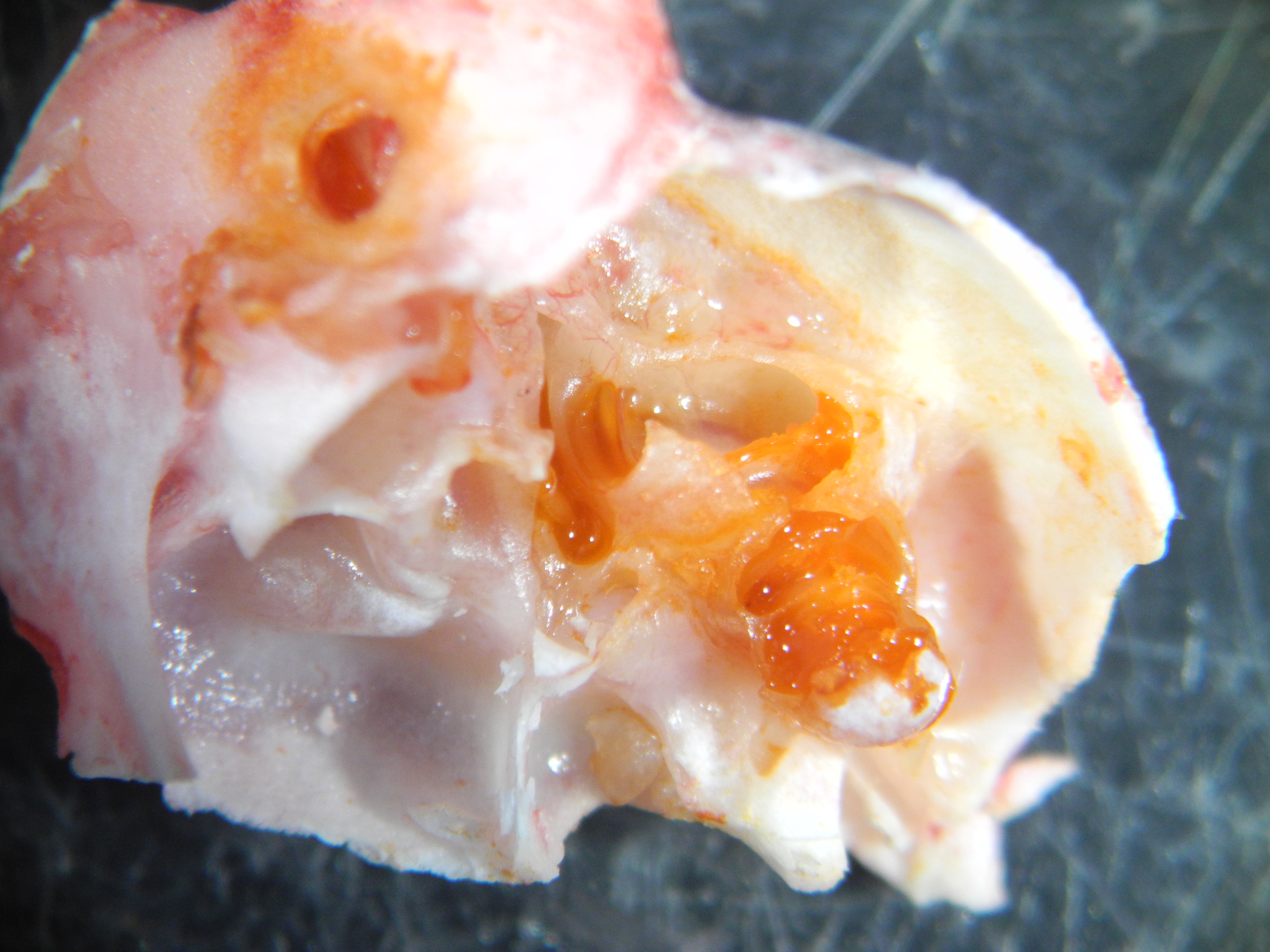

Supplement: Supplemental Information 6 [file peerj-11-15562-s006.jpg]

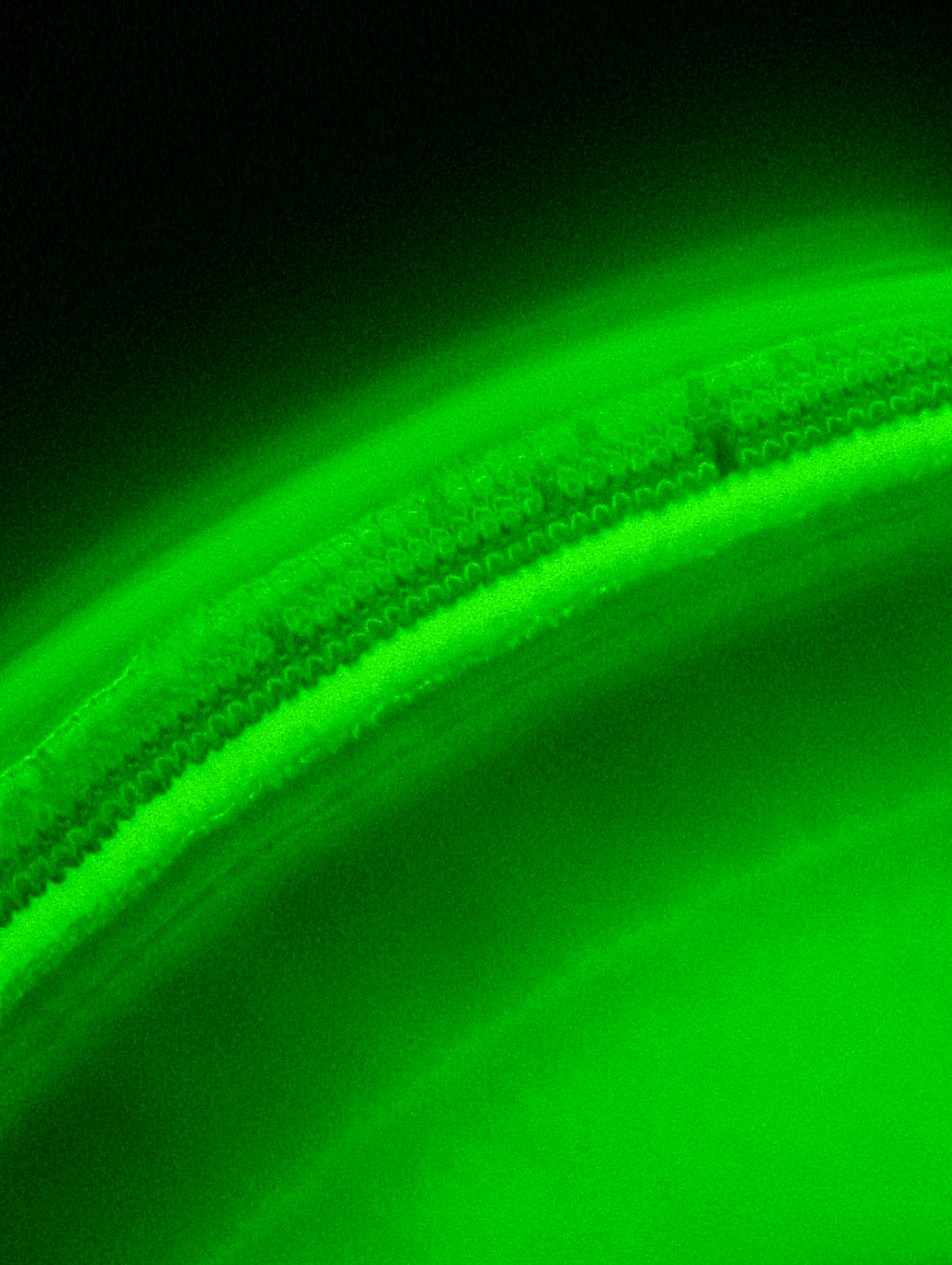

Supplement: Supplemental Information 7 [file peerj-11-15562-s007.bmp]

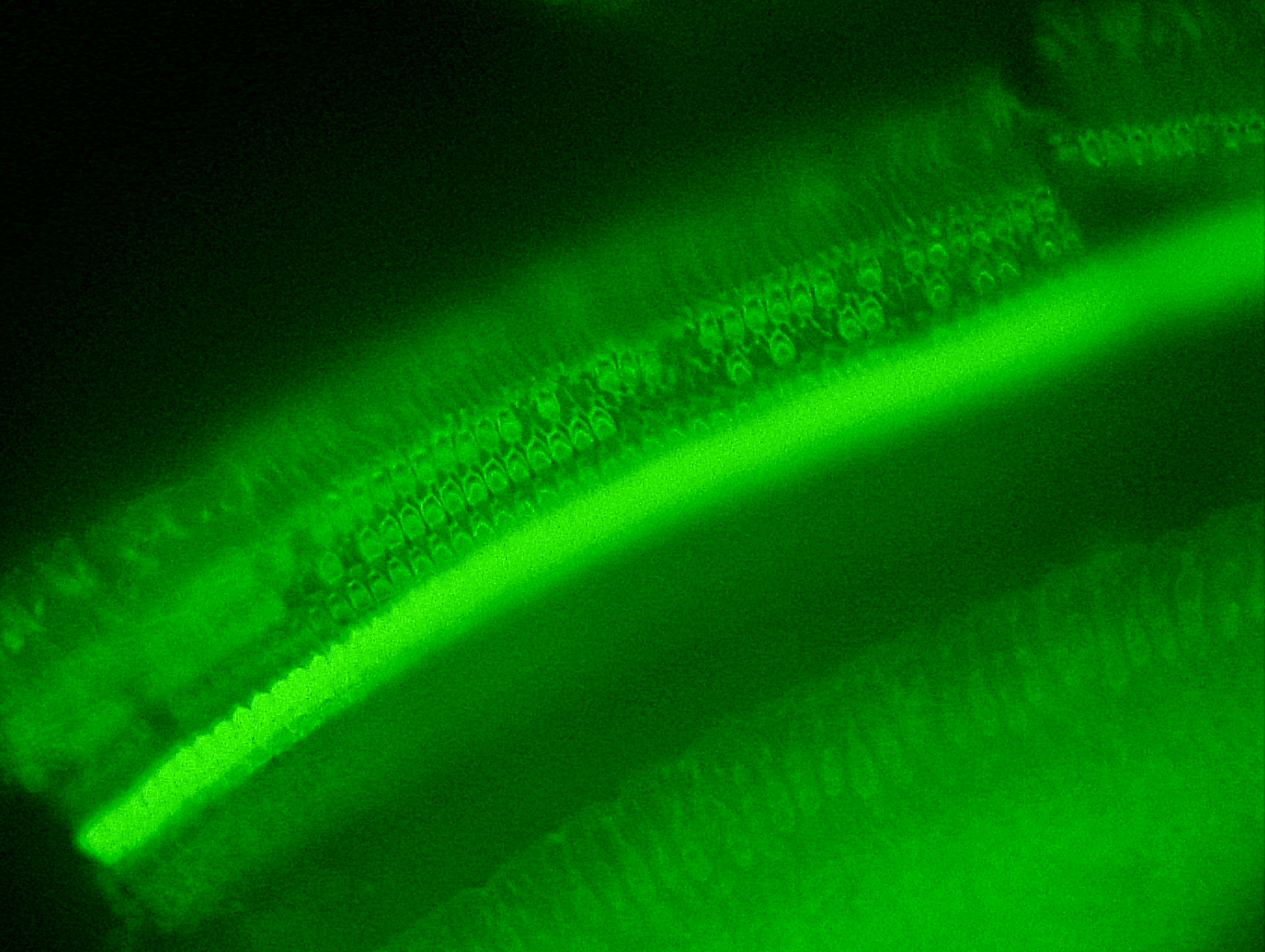

Supplement: Supplemental Information 8 [file peerj-11-15562-s008.bmp]
